# Supplementary material for: Electrospun Scaffolds Containing Silver-Doped Hydroxyapatite with Antimicrobial Properties for Applications in Orthopedic and Dental Bone Surgery
Source: J Funct Biomater. 2020 Aug 14;11(3):58. doi: 10.3390/jfb11030058 (PMC7563183; doi:10.3390/jfb11030058)
Supplement: Supplementary file 1 [file jfb-11-00058-s001.pdf]

Supplementary Materials

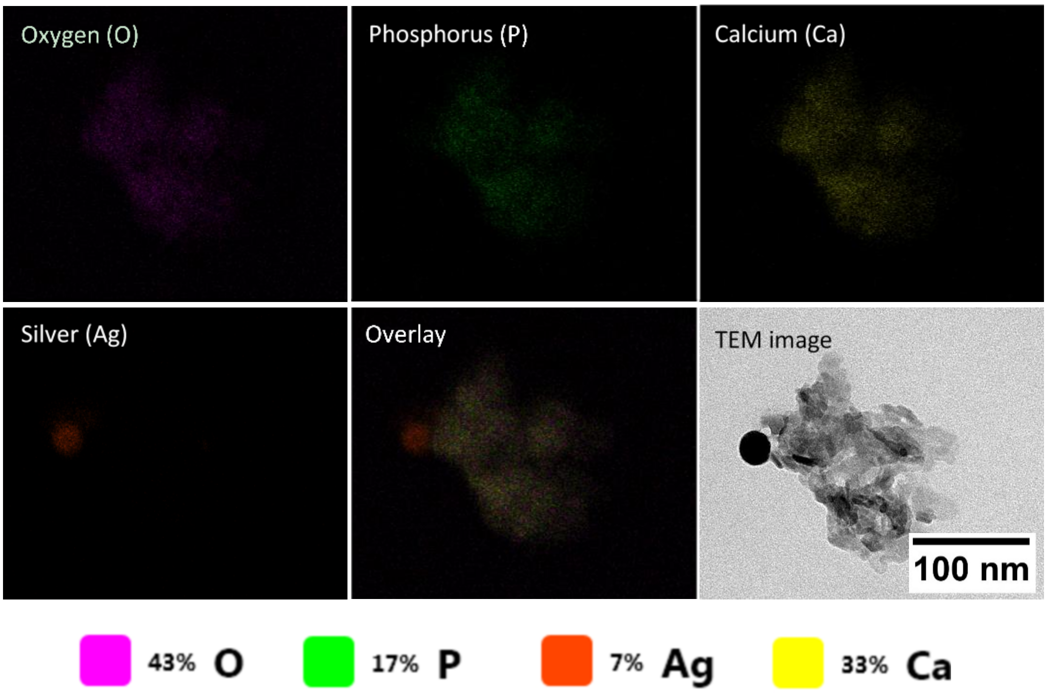

Figure S1. EDX elemental mapping of 10 Ag mol.% samples detecting oxygen, phosphorus, calcium and silver. TEM image of the analysed material is also shown for comparison along with an overlay image of all elements in a single frame.
